# Stability of the Human Hsp90-p50^Cdc37^ Chaperone Complex against Nucleotides and Hsp90 Inhibitors, and the Influence of Phosphorylation by Casein Kinase 2

**DOI:** 10.3390/molecules20011643

**Published:** 2015-01-19

**Authors:** Sanne H. Olesen, Donna J. Ingles, Jin-Yi Zhu, Mathew P. Martin, Stephane Betzi, Gunda I. Georg, Joseph S. Tash, Ernst Schönbrunn

**Affiliations:** 1 Drug Discovery Department, H. Lee Moffitt Cancer Center and Research Institute, Tampa, FL 33612, USA; E-Mails: sanne.olesen@agilent.com (S.H.O.); Donna.Ingles@moffitt.org (D.J.I.); Jinyi.Zhu@moffitt.org (J.-Y.Z.); Mathew.Martin@newcastle.ac.uk (M.P.M.); stephane.betzi@inserm.fr (S.B.); 2 Department of Medicinal Chemistry, College of Pharmacy, University of Minnesota, Minneapolis, MN 55414, USA; E-Mail: georg@umn.edu; 3 Department of Molecular and Integrative Physiology, University of Kansas Medical Center, Kansas City, KS 66160, USA; E-Mail: JTASH@kumc.edu

**Keywords:** heat shock proteins, protein–protein interactions, protein phosphorylation, protein kinase, small molecule inhibitors

## Abstract

The molecular chaperone Hsp90 is regulated by co-chaperones such as p50^Cdc37^, which recruits a wide selection of client protein kinases. Targeted disruption of the Hsp90-p50^Cdc37^ complex by protein–protein interaction (PPI) inhibitors has emerged as an alternative strategy to treat diseases characterized by aberrant Hsp90 activity. Using isothermal microcalorimetry, ELISA and GST-pull down assays we evaluated reported Hsp90 inhibitors and nucleotides for their ability to inhibit formation of the human Hsp90β-p50^Cdc37^ complex, reconstituted *in vitro* from full-length proteins. Hsp90 inhibitors, including the proposed PPI inhibitors gedunin and H2-gamendazole, did not affect the interaction of Hsp90 with p50^Cdc37^ *in vitro*. Phosphorylation of Hsp90 and p50^Cdc37^ by casein kinase 2 (CK2) did not alter the thermodynamic signature of complex formation. However, the phosphorylated complex was vulnerable to disruption by ADP (IC_50_ = 32 µM), while ATP, AMPPNP and Hsp90 inhibitors remained largely ineffective. The differential inhibitory activity of ADP suggests that phosphorylation by CK2 primes the complex for dissociation in response to a drop in ATP/ADP levels. The approach applied herein provides robust assays for a comprehensive biochemical evaluation of potential effectors of the Hsp90-p50^Cdc37^ complex, such as phosphorylation by a kinase or the interaction with small molecule ligands.

## 1. Introduction

The 90 kDa heat shock protein (Hsp90) is a highly conserved molecular chaperone that activates or protects client proteins in an ATP-dependent manner. The two cytosolic homologues of human Hsp90 (α and β) have a sequence similarity of more than 85%, but little is known about their differences in function [1,2]. Hsp90 and its co-chaperones have been associated with various types of cancer [3,4,5], Alzheimer’s and other neurodegenerative diseases [6], viral diseases [7] and vascular diseases [8]. The specificity of Hsp90 towards more than 300 different client proteins is controlled by a variety of co-chaperones, including p50^Cdc37^ [9]. p50^Cdc37^ is involved in the recruitment of more than 45 different kinases to Hsp90, resulting in their subsequent activation, and many of these client kinases have been implicated in oncogenic pathways [4]. Silencing of p50^Cdc37^ in human colon and breast cancer cell lines destabilizes many of these kinases *in vivo*, leading to decreased proliferation and increased sensitivity of these cell types to Hsp90 inhibitors [10].

During acute heat shock, the phosphorylation of Hsp90 significantly increases [11]. Several kinases are known to phosphorylate Hsp90, the most prominent of which are casein kinase II (CK2) [12] and Wee1 [13,14]. CK2 phosphorylates several serine and threonine residues in Hsp90 and in co-chaperone proteins such as p50^Cdc37^and p23, constituting an important but complex regulatory component of chaperone function in eukaryotic cells [15,16]. CK2 phosphorylates Hsp90β at multiple residues [12,15], affecting its ability to interact with client proteins such as Apaf-1 and AhR [17,18]. Among the residues phosphorylated by CK2 are Ser226 and Ser255, located at a distinctive charged linker region between the N-terminal and the middle domain of Hsp90, the structure and function of which is obscure. Deletion of this charged linker decreases the ability of Hsp90 proteins to function as chaperones in the cell [19,20]. Phosphorylation of p50^Cdc37^ at residue Ser13 by CK2 affects the recruitment of client kinases to the Hsp90-p50^Cdc37^ complex [21,22]. In addition to the phosphorylation by CK2 and Wee1, Hsp90 is also subject to phosphorylation by v-Src [23].

Several small molecule inhibitors of Hsp90 have been developed as potential therapeutic agents. NVP-AUY922 is currently in Phase II clinical trials for non-small cell lung cancer (NSCLC) [5,24,25], Ganetespib is in Phase III combination therapy trials with docetaxel for NSCLC, while Phase III trials with Retaspimycin (IPI-504) and Tanespimycin (17-AAG) have been terminated or halted (www.clinicaltrials.gov). The majority of reported and validated Hsp90 inhibitors exclusively target the ATP site of the N-terminal domain of Hsp90. However, the association of Hsp90 with co-chaperones has stimulated efforts towards the development of protein–protein interaction (PPI) inhibitors. Non-hydrolysable ATP analogues were reported to disrupt complex formation between Hsp90β and p50^Cdc37^, while ADP, geldanamycin, and the geldanamycin derivatives 17-AAG and 17-DMAG showed no effect [26]. It was recently suggested that Hsp90 is a molecular target of the male contraceptive drug candidate H2-gamendazole (H2-GMZ) [27,28], and it was concluded that H2-GMZ shares a similar mode of action as the proposed PPI inhibitor gedunin by inhibiting formation of the Hsp90-p50^Cdc37^ complex [29].

Here we evaluated the ability of reported Hsp90 inhibitors and nucleotides to disrupt the interaction of human full-length Hsp90β and p50^Cdc37^ using isothermal titration calorimetry (ITC), ELISA, and GST pull-down assays. We show that previously reported PPI inhibitors of the Hsp90-p50^Cdc37^ complex lack binding potential or inhibitory activity against the complex. Among the compounds tested only ADP effectively disrupted the complex, particularly upon phosphorylation by CK2.

## 2. Results and Discussion

### 2.1. In Vitro Reconstitution of the Human Hsp90β-p50^Cdc37^ Complex and Phosphorylation by CK2

**Figure 1:**
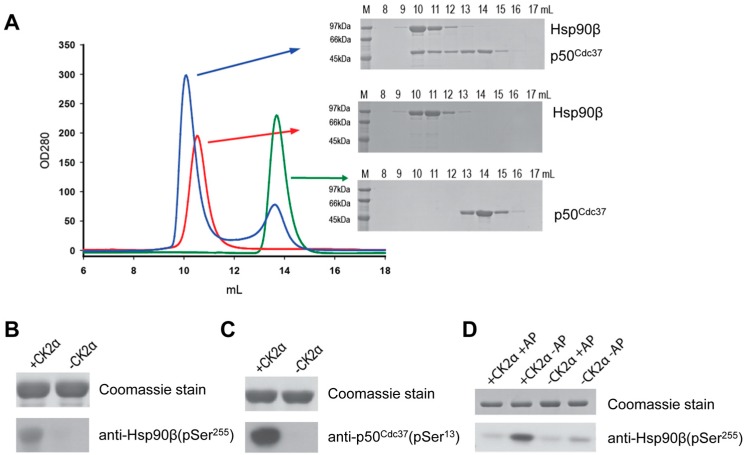
*In vitro* reconstitution of the human full-length Hsp90β:p50^Cdc37^ complex and phosphorylation by CK2α. (**A**) Elution profiles and corresponding SDS-PAGE for analytical gel filtration experiments showing complex formation (blue) upon incubation of free Hsp90β (red) with free p50^Cdc37^ (green). (**B**) Coomassie-stained SDS-PAGE and Western blot analysis of purified Hsp90β in the presence and absence of CK2α and ATP using anti-Hsp90β (pSer^255^); (**C**) Coomassie-stained SDS-PAGE and Western blot analysis of purified p50^Cdc37^ in the presence and absence of CK2α and ATP using anti-p50^Cdc37^ (pSer^13^); (**D**) Phosphorylation states of Ser255 in Hsp90β upon phosphorylation by CK2α and dephosphorylation by alkaline phosphatase (AP).

The ability of human p50^Cdc37^ to interact with human Hsp90 was assessed using full-length Hsp90β, a deletion mutant of Hsp90β (denoted ΔHsp90) which lacks the charged linker region residues 220–275, and the N-terminal domain of Hsp90β (denoted N-Hsp90). All proteins were purified to homogeneity, and size-exclusion chromatography confirmed that Hsp90 and p50^Cdc37^ eluted as dimeric proteins. Addition of p50^Cdc37^ to Hsp90β resulted in the formation of a stable complex with 1:1 stoichiometry as judged by SDS-PAGE (Figure 1A). Full-length Hsp90β is subject to phosphorylation by CK2 at residues Ser226 and Ser255 of the charged linker region [12], and p50^Cdc37^ is phosphorylated by CK2 at residue Ser13 [21]. In order to evaluate the effect of phosphorylation on the formation and stability of the Hsp90-p50^Cdc37^ complex, we produced recombinant human CK2α for the quantitative phosphorylation of both Hsp90 and p50^Cdc37^. Phosphorylation of Ser255 of Hsp90β and Ser13 of p50^Cdc37^ was assessed by Western blotting using phospho-specific antibody (Figure 1B–D). Attempts to verify pSer226 of Hsp90β failed due to the lack of specificity of commercially available antibodies against pSer226.

**Figure 2:**
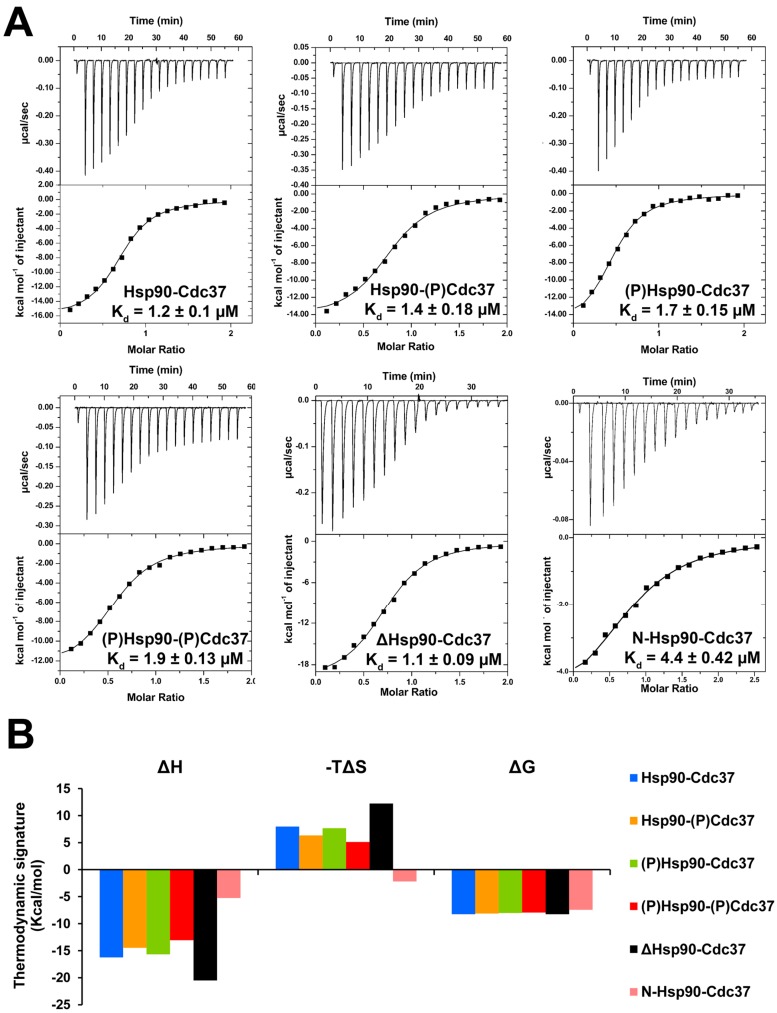
Influence of phosphorylation by CK2 on the thermodynamics of Hsp90β-p50^Cdc37^ complex formation. (**A**) Isothermal titration calorimetry (ITC) experiments of the Hsp90β-p50^Cdc37^ complex formation using unphosphorylated and phosphorylated proteins. The last two graphs show the interactions of Hsp90 without the acidic loop (ΔHsp90) and of the N-terminal domain (N-Hsp90); (**B**) Thermodynamic signatures of the protein–protein interactions (PPI).

The thermodynamic profiles of the complex formation between p50^Cdc37^ and Hsp90β proteins, and the effect of phosphorylation by CK2 were determined by direct binding studies using ITC (Figure 2A). With dissociation constants between 1.1 and 1.9 µM no significant differences in binding affinities were observed for the interaction of the different Hsp90 proteins with p50^Cdc37^. Only the N-terminal domain of Hsp90 displayed a slightly decreased binding potential (*K*_d_ = 4.4 µM). The *K*_d_ values are similar to the reported dissociation constant of 1.5 µM for the interaction of yeast Hsp82 with human p50^Cdc37^ [30]. Notably, phosphorylation of Hsp90 or p50^Cdc37^ did not appear to impact complex formation; the thermodynamic signatures were similar for phosphorylated and unphosphorylated proteins (Figure 2B). Complex formation is characterized by favourable enthalpy and unfavourable entropy contributions, indicative of substantial conformational changes in one or both proteins upon complex formation. The deletion mutant displayed an increase in both enthalpy and entropy, and the N-terminal domain appeared to interact with p50^Cdc37^ through a more favourable change in entropy, albeit at the expense of a significantly reduced enthalpy. These data suggest the involvement of regions beyond the N-terminal domain that undergo structural rearrangements upon complex formation.

### 2.2. Effect of Phosphorylation on the Interaction of Hsp90β with Nucleotides and Inhibitors

**Figure 3:**
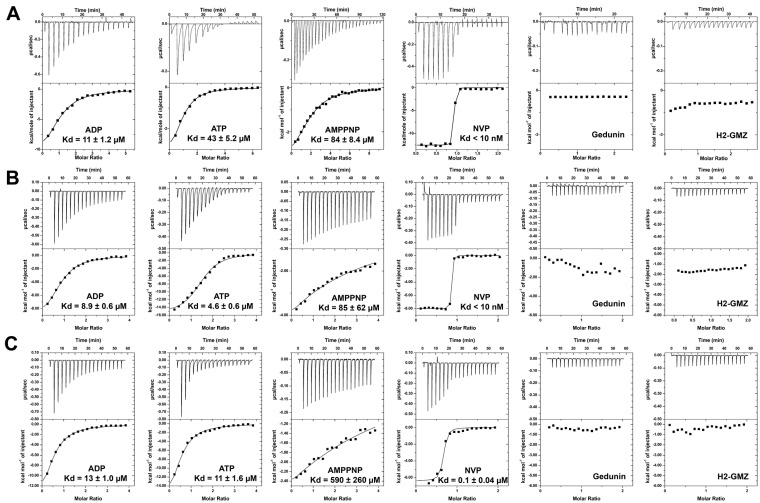
Interaction of Hsp90β proteins with nucleotides and reported inhibitors determined by ITC. Shown are the results of titrations experiments for small molecule ligands with ΔHsp90β (**A**); full-length Hsp90β (**B**); and CK2-phosphorylated Hsp90β (**C**).

The influence of phosphorylation by CK2 on the binding potential of nucleotides and small molecule inhibitors was evaluated in a series of ITC experiments (Figure 3). Interaction with ADP was similar for unphosphorylated and phosphorylated Hsp90 and ΔHsp90 with *K*_d_ values between 9 and 13 µM, but significant changes were observed for ATP and AMP-PNP. The full-length protein interacted equally well with ADP and ATP, irrespective of its phosphorylation state, whereas deletion of the charged linker reduced the binding potential for ATP significantly (*K*_d_ = 43 µM). With *K*_d_ values of 84 and 85 µM, AMP-PNP was a relatively weak binder of ΔHsp90 and the full-length protein, and phosphorylation further reduced its binding potential (*K*_d_ > 500 µM). Similarly, the potent ATP site-directed inhibitor NVP-AUY922 interacted tightly with ΔHsp90β and unphosphorylated full-length protein (*K*_d_ < 10 nM), but phosphorylation appeared to reduce the binding affinity (*K*_d_ = 0.1 µM). The data suggest that phosphorylation of the charged linker region influences the interaction of Hsp90 with ATP site-directed ligands. The dissociation constants determined here for unphosphorylated human Hsp90β correlate well with previously reported values for Hsp90 proteins, with the exception of ATP (Table 1). Conspicuously, the recently proposed Hsp90 inhibitors gedunin and H2-GMZ failed to display binding potential towards Hsp90β (Figure 3). None of the nucleotides and inhibitors interacted with p50^Cdc37^ (data not shown).

**Table 1:** Dissociation constants determined by ITC and comparison with reported values.

Binding Partner/Ligand	Hsp90	Observed K_d_ (µM)	Reported K_d_ (µM)	Reference
p50^Cdc37^	yHsp82-FL	n/d	1.46	[30]
	hΔHsp90β	1.1	n/a	
	hHsp90β-N	4.4	n/a	
	hHsp90β-FL	1.2	n/a	
	h(p)Hsp90β-FL	1.7	n/a	
(p)p50^Cdc37^	hHsp90β-FL	5.1	n/a	
	h(p)Hsp90β-FL	8.6	n/a	
ADP	hΔHsp90β	11	n/a	
	hHsp90β-FL	8.9	7.2	[31]
	h(p)Hsp90β-FL	13	n/a	
ATP	hΔHsp90β	43	n/a	
	hHsp90β-FL	4.6	240	[31]
	h(p)Hsp90β-FL	11	n/a	
AMP-PNP	hΔHsp90β	84	n/a	
	hHsp90β-FL	85	148	[31]
	h(p)Hsp90β-FL	590	n/a	
NVP-AUY922	hΔHsp90β	<0.01	n/a	
	hHsp90β-FL	<0.01	0.0017	[32]
	h(p)Hsp90β-FL	0.1	n/a	
17-DMAG	hHsp90β-FL	n/d	0.35	[33]
	hΔHsp90β	0.79	n/a	
Radicicol	yHsp82-FL	n/d	0.019	[34]
	hΔHsp90β	<0.01	n/a	

### 2.3. Evaluation of Small Molecules as PPI Inhibitors of the Hsp90β-p50^Cdc37^ Complex

**Figure 4:**
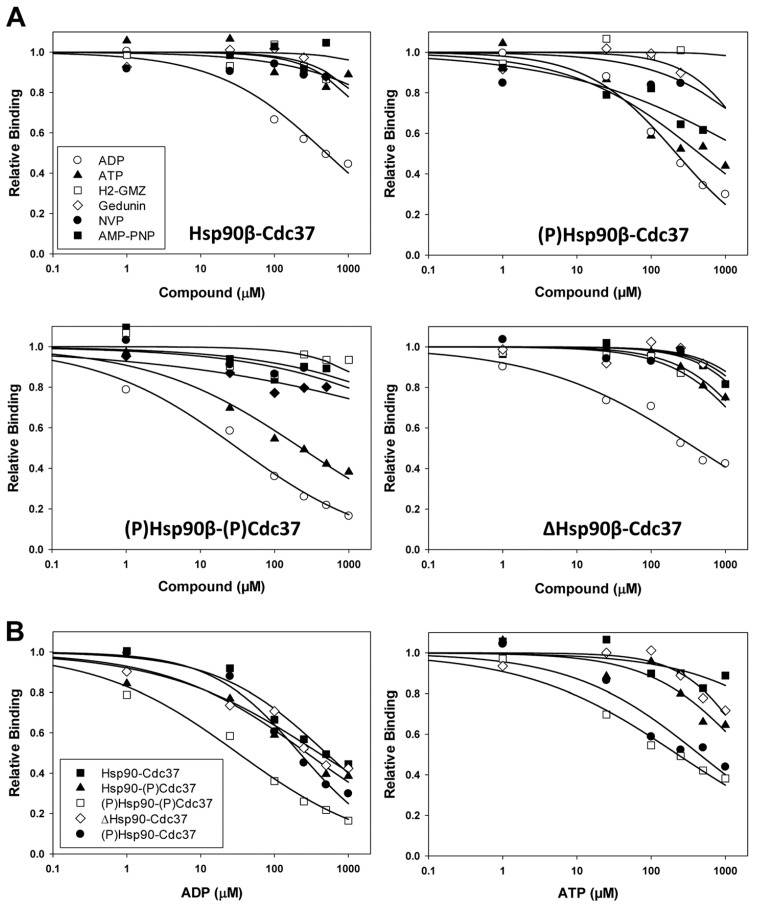
Effect of nucleotides and inhibitors on the Hsp90β-p50^Cdc37^ complex as determined by ELISA. (**A**) Dose-response experiments for the complex formation between immobilized GST-p50^Cdc37^ and Hsp90β (unphosphorylated or phosphorylated) in the presence of ADP, ATP, AMP-PNP, NVP-AUY922, gedunin, or H2-GMZ, as indicated; (**B**) Replot of (A) to compare the effect of phosphorylation on the stability of the complex. The Hsp90-(p)p50^Cdc37^ complex was probed only against ADP and ATP but not against inhibitors. IC_50_ values are shown in Table 2.

Next, we developed a robust ELISA assay using immobilized GST-p50^Cdc37^ to determine the effect of nucleotides and inhibitors on complex formation with Hsp90β using unphosphorylated and CK2-phosphorylated proteins (Figure 4 and Table 2). Among the compounds tested, only ADP displayed appreciable disruptive potential against the unphosphorylated complex with IC_50_ values between 400 and 500 µM. The inhibitory activity of ADP slightly increased upon phosphorylation of either Hsp90 or p50^Cdc37^ (IC_50_ = 220–270 µM), while phosphorylation of both proteins significantly increased sensitivity of the complex towards ADP (IC_50_ = 32 µM). ATP was largely ineffective against the unphosphorylated complex (IC_50_ > 1 mM), while the phosphorylated complex was inhibited by ATP with an IC_50_ value of 230 µM. AMP-PNP and NVP-AUY922, along with gedunin and H2-GMZ, did not show appreciable inhibitory activity against the unphosphorylated or phosphorylated complex. These results indicate that ADP is a remarkably potent and differential inhibitor of the interaction between phosphorylated Hsp90β and p50^Cdc37^.

**Table 2:** Disruptive potential of small molecules against Hsp90β-p50^Cdc37^ complexes determined by ELISA.

Complex	IC_50_ (µM)						
	ADP	ATP	AMP-PNP	H2-GMZ	H2-GMZ	Gedunin	NVP
Hsp90-p50^Cdc37^	500 ± 94	>1000	>1000	>1000	>1000	>1000	>1000
(p)Hsp90-p50^Cdc37^	220 ± 24	450 ± 150	>1000	>1000	>1000	>1000	>1000
Hsp90-(p)p50^Cdc37^	270 ± 35	>1000	n/d	n/d	n/d	n/d	n/d
(p)Hsp90-(p)p50^Cdc37^	32 ± 5.6	230 ± 40	>1000	>1000	>1000	>1000	>1000
ΔHsp90-p50^Cdc37^	400 ± 81	>1000	>1000	>1000	>1000	>1000	>1000

### 2.4. ADP Exerts Inhibitory Activity through Binding to the ATP Site in the N-Terminal Hsp90 Domain

**Figure 5:**
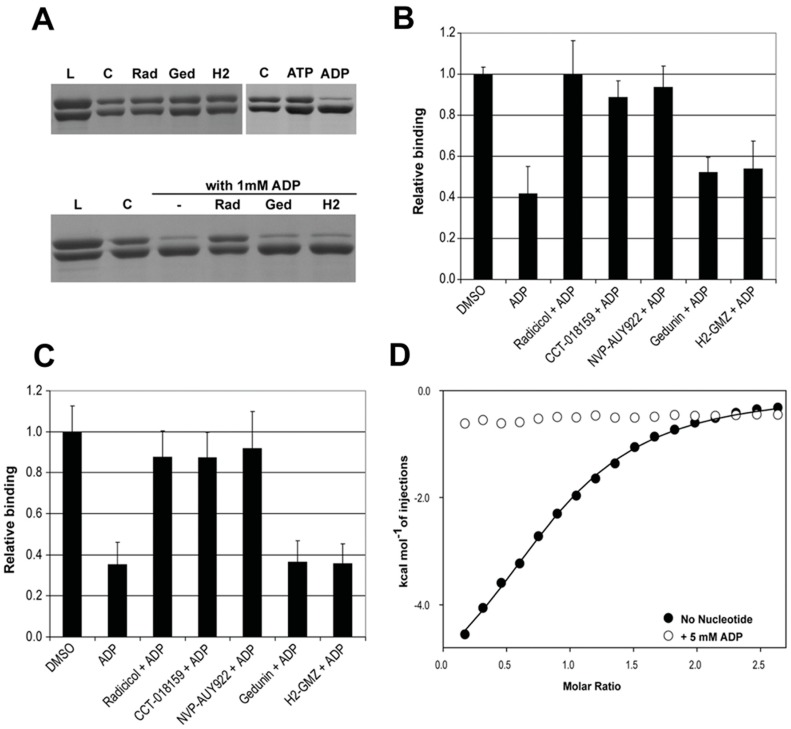
The PPI inhibitory activity of ADP proceeds through binding to the ATP site of Hsp90. (**A**) SDS-PAGE of GST pull-down experiments for ΔHsp90β interaction with GST-p50^Cdc37^. The two proteins were incubated with 1 mM nucleotide or 0.1 mM inhibitor for 15 min, loaded onto a GST-affinity column, and eluted with reduced glutathione. The upper gels show elution of a stable complex in the presence of inhibitors or ATP, but the presence of ADP caused disruption of the complex. The bottom gel shows the result of a competition experiment in which the presence of 0.1 mM radicicol protected the complex from ADP, whereas gedunin and H2-GMZ had no effect. L = load, C = control (no ligand), Rad = radicicol, Ged = gedunin, H2 = H2-GMZ; (**B**,**C**) Competition experiments for the interaction of ΔHsp90β (B) or ΔHsp90α (C) with p50^Cdc37^ in the presence of 1 mM ADP and 0.25 mM inhibitors (0.1 mM for gedunin) determined by ELISA. Only ATP site-directed inhibitors protected the complex from ADP; (**D**) Binding isotherms for the interaction of the N-terminal domain of Hsp90β with p50^Cdc37^ in the absence and presence of ADP.

To assess if the inhibitory activity of ADP is a result of binding to the ATP site, we performed competition experiments by GST-pull down and ELISA using radicicol as a representative ATPase inhibitor of Hsp90, in parallel with gedunin and H2-GMZ. Under these conditions, only ADP inhibited formation of the complex. The presence of radicicol completely protected the complex from ADP, indicating competition for binding to the same site (Figure 5A). As expected from the ITC experiments, gedunin and H2-GMZ were ineffective. ELISA assays confirmed that ATP site-directed Hsp90 inhibitors ameliorated the effect of ADP for both ΔHsp90β and ΔHsp90α (Figure 5B,C). Finally, ITC experiments using the N-terminal domain of Hsp90β revealed that its interaction with p50^Cdc37^ was abolished in the presence of 5 mM ADP (Figure 5D). Combined, the data suggest that the PPI inhibitory activity of ADP is a result of binding to the ATP site in the N-terminal domain of Hsp90.

### 2.5. Possible Structural Basis for the PPI Inhibitory Activity of ADP 

**Figure 6:**
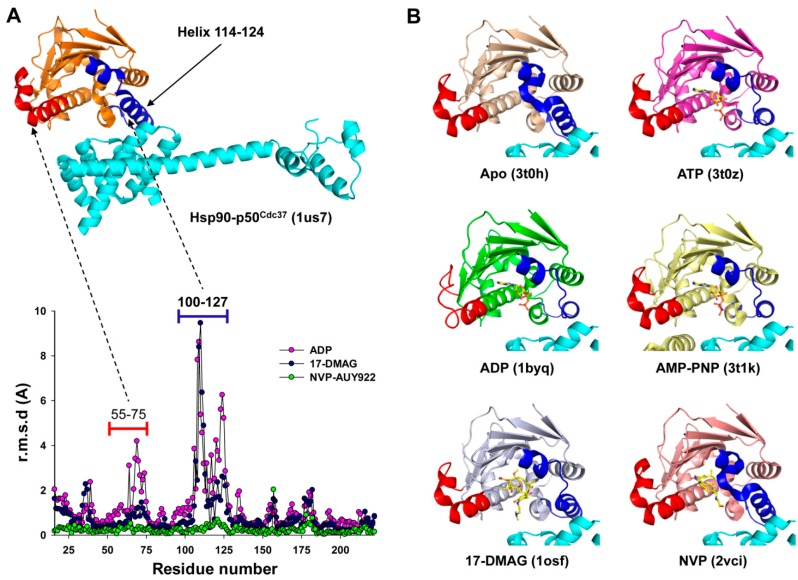
ADP-induced structural changes in Hsp90 may impact complex formation with p50^Cdc37^. (**A**) Root mean square deviation (r.m.s.d.) analysis of the N-terminal domain of human Hsp90α in the ligand-free state (3T0H) against the liganded states with ADP (1BYQ), 17-DMAG (1OSF), or NVP-AUY922 (2VCI). Residues 100-127 and, to a lesser extent, residues 55-75, undergo large structural changes upon ligand binding, corresponding to the blue and red highlighted regions of the yeast Hsp90:human p50^Cdc37^ complex (1US7) shown at the top of the graph. p50^Cdc37^ is shown in cyan; (**B**) Superposition of different liganded states of the N-terminal domain of Hsp90 show conformational changes that may impact the interaction with p50^Cdc37^.

To explore potential structural changes in Hsp90 uniquely induced by ADP, we compared a set of crystal structures available for human N-terminal Hsp90α (unliganded: PDB 3T0H [35], ADP: PDB 1BYQ [36], ATP: PDB 3T0Z [35], AMP-PNP: PDB 3T1K [35], 17-DMAG: PDB 1OSF [37], NVP-AUY922: PDB 2VCI [38]). Substantial structural changes occur in Hsp90 upon ligand binding, particularly near residues 100–127, with root mean square deviations (r.m.s.d.) up to 10 Å (Figure 6A). The only crystal structure available for the Hsp90-p50^Cdc37^ complex is that of yeast N-terminal Hsp82, void of nucleotides, with a C-terminal construct of human p50^Cdc37^ [30]. Superposition of this complex with the aforementioned structures of liganded N-terminal Hsp90 illustrates that, among the structurally variable regions, only helix 114–124 directly interacts with p50^Cdc37^. This helix adopts distinctly different conformations, depending on the type of ligand bound to the ATP site (Figure 6B). The nucleotide-liganded states share a similar helix conformation which differs significantly from the conformation adopted upon interaction with p50^Cdc37^. It is conceivable that stabilization of the helix conformation in the nucleotide liganded states negatively impacts the interaction with p50^Cdc37^. The structural basis for the high inhibitory activity of ADP relative to ATP or AMP-PNP is less obvious and will require crystal structures of the full-length human Hsp90-p50^Cdc37^ complex liganded with nucleotides or ATPase inhibitors. For the known yeast Hsp90-p50^Cdc37^ structure, the protein–protein interface in the immediate vicinity of the ATP site suggests that the Arg167 side chain of p50^Cdc37^ may establish a salt bridge with the terminal phosphate group of ATP or AMP-PNP, but not with ADP. The inability of ADP to interact with this arginine residue may explain the differential inhibitory activity of ADP *versus* ATP.

## 3. Experimental Section

### 3.1. Materials

All reagents were purchased from Sigma-Aldrich unless otherwise noted. Radicicol and gedunin were purchased from Tocris Bioscience. NVP-AUY922 and 17-DMAG were purchased from Selleck Chemicals. H2-GMZ was synthesized as previously described [27]. Stock solutions were freshly prepared at 100 mM concentration in water (nucleotides) or in 100% DMSO (inhibitors) and were stored in 0.1 mL aliquots at −80 °C. Protein concentration was determined by A280 molar absorbance using a Nanodrop ND-1000 spectrophotometer.

### 3.2. Cloning and Expression

The gene encoding human full-length Hsp90β was subcloned from T47D cell line cDNA, and human full-length CK2α was subcloned from MCF7 cell line cDNA, both a kind gift from Dr. Jonathan Lancaster, Moffitt Cancer Center. The genes encoding the deletion mutants human Hsp90α (1-732 Δ226-283), human Hsp90β (1-724 Δ220-275), human N-terminal Hsp90β (1-219, denoted Hsp90β-N), and human full-length p50^Cdc37^ (residues 1-378) were synthesized by GeneArt AG with optimized codon usage for *E. coli* expression. The deleted sections in the two Hsp90 genes were each replaced by four glycine residues. Hsp90α and Hsp90β were subcloned into the pET28a vector (Novagen), and p50^Cdc37^ was subcloned into a modified pET28a vector containing a maltose-binding protein (MBP) solubility tag and a PreScission protease cleavage site using restriction enzymes BamHI and NotI (Fermentas) and T4 DNA ligase (Invitrogen) following the manufacturers’ instructions. BamHI was substituted with NdeI for full-length Hsp90β. p50^Cdc37^ was also subcloned into the pGEX6P-1 vector (GE Healthcare) using the same procedure as above to provide an N-terminal GST-tag for GST pull-down and ELISA experiments. The plasmids were transformed into E. coli BL21 Star (DE3) (Hsp90α and Hsp90β, Invitrogen), Tuner (DE3) (p50^Cdc37^ modified pET28a, Novagen), or BL21 (DE3) (p50^Cdc37^ pGEX6P-1, Stratagene) expression strains. Transformed cells were grown in LB broth at 37 °C until the OD_600_ reached 0.4, then the temperature was lowered to 16 °C, and cells were induced with 0.1 mM IPTG at OD_600_ = 0.5–0.7. Cultures were then grown for 20–24 h before harvesting by centrifugation (30 min at 30,000× *g*, 4 °C).

### 3.3. Protein Purification

Harvested cells containing overexpressed proteins were resuspended in 50 mM Tris buffer (pH 8.0 at 4 °C) containing 300 mM NaCl, 10 mM imidazole, 0.1 mg·mL^−1^ lysosyme, and 0.01% Triton X-100 at 4 °C for 2 h. After sonication and centrifugation (35 min at 35,000× *g*), the target proteins were purified by FPLC using a combination of nickel affinity, anion exchange, and size-exclusion chromatography, incorporating an additional step to cleave the His_6_-MBP-tag from p50^Cdc37^ and CK2α using PreScission protease. GST-tagged p50^Cdc37^ and GST alone (as a control for ELISA studies) were purified using glutathione sepharose and size exclusion chromatography by FPLC. Analytical size exclusion chromatography was performed with a Superdex200 10/300 GL column at room temperature using a flow rate of 0.3 mL/min. Samples contained 85 µM protein in 50 mM HEPES (pH 7.5), 50 mM KCl, 5 mM MgCl_2_, and 1 mM DTT. Eluted protein was collected in 1 mL fractions and analyzed on 12% SDS-PAGE gels stained with Coomassie blue for visualization.

### 3.4. GST Pull-Down

GST pull-down experiments were carried out using 1 mL GST 4 FastFlow resin in 8 mL Flex columns (Kontes). All experiments were performed at room temperature by gravity flow. Loading and wash buffers contained 50 mM Tris pH 8, 50 mM KCl, 5 mM MgCl_2_, and 5% DMSO. The elution buffer contained an additional 10 mM reduced glutathione. Protein/compound mixtures containing equimolar amounts of each protein (25 μM), as well as 1 mM nucleotide and/or 0.25 mM compound (0.1 mM gedunin due to solubility issues), in a 1 mL total volume were incubated for 15 min at room temperature prior to loading. The resin was washed with 20 CV wash buffer, and samples were eluted with 2 CV elution buffer. Eluted protein was analysed on 12% SDS-PAGE stained with Coomassie blue for visualization.

### 3.5. In Vitro Phosphorylation

Phosphorylation of full-length Hsp90β and p50^Cdc37^ was performed using purified full-length CK2α. The reaction was carried out in a 2.5 mL volume containing 50 µM Hsp90β or p50^Cdc37^, 0.5 µM CK2α, 0.5 mM ATP, 20 mM Tris (pH 7.5), 50 mM KCl, and 10 mM MgCl_2_, and was incubated for 1 h at 30 °C. Samples were removed for analysis of phosphorylation status, and the proteins were re-buffered into 50 mM HEPES (pH 7.5), 150 mM NaCl, and 1 mM DTT (ELISA), or into 50 mM HEPES (pH 7.5) for ITC, using PD-10 gravity columns (GE Healthcare). Dephosphorylation was performed using 2.5 units of FastAP (alkaline phosphatase from Thermo Scientific) in buffer containing 10 mM Tris (pH 8.0 at 37 °C), 5 mM MgCl_2_, 100 mM KCl, 0.02% Triton-X 100, and 0.5 mg/mL BSA for 1 h at 37 °C.

### 3.6. SDS-PAGE and Western Blot Analysis

Samples were run on 12% SDS-PAGE gels at 120 V and 4 °C for 1.5 h. The gels were then either stained with Coomassie Blue at room temperature or transferred to a PVDF membrane in a wet-transfer Mini Trans-Blot Cell (Bio-Rad) following the manufacturer’s instructions. The membrane was blocked with 5% skim milk in 1× TBS-T (50 mM Tris pH 8, 150 mM NaCl, 0.1% (*v*/*v*) Tween-20) and rinsed with 1× TBS-T. The membrane was incubated overnight at 4 °C with anti-p50^Cdc37^ phosho-pS13 rabbit mAb (Epitomics, diluted to 0.04 μg/mL) or anti-Hsp90β phospho-pS255 rabbit pAb (Abcam, diluted to 2 µg/mL) in SuperBlock + 0.01% Tween-20 (Thermo Scientific, Rockford, IL, USA), and then incubated with anti-rabbit horseradish peroxidase (HRP)-conjugated IgG secondary antibody (Cell Signaling Technology) for 2 h at room temperature. Western blots were developed using HyGLO Quick Spray HRP substrate and exposed on HyBlot CL autoradiography film (Denville Scientific).

### 3.7. ELISA Microtiter Plate Assay

Purified GST-p50^Cdc37^ (4 µM) or control (purified GST alone, 4 µM) was added to glutathione-coated 96-well plates (Pierce) in 100 μL protein buffer (50 mM HEPES, pH 7.5, 150 mM NaCl, 10 mM MgCl_2_, 0.05% Tween-20, and 0.1% BSA) and incubated for 1 h at room temperature (triplicate wells per sample). Wells were washed three times with 1× HBS-T (50 mM HEPES pH 7.5, 150 mM NaCl, 0.05% Tween-20) and blocked with 250 μL 5% BSA (*w*/*v*) and 10 mM MgCl_2_ in 1× HBS-T for 1 h at room temperature before washing again three times with 1× HBS-T. Purified His_6_-Hsp90 protein (4 μM) was pre-incubated in protein buffer with nucleotides and/or small molecule inhibitors in the presence of 5% DMSO for 30 min at room temperature (the presence of DMSO was not found to significantly affect the protein–protein interactions). The mixtures were then added to the wells (100 μL per well, in triplicates) and incubated for 1 h at room temperature before washing. The wells were incubated with anti-His_6_-tag mAb (1 mg/mL stock, Abcam), diluted 1:2000 in antibody buffer (50 mM HEPES pH 7.5, 150 mM NaCl, 10 mM MgCl_2_, 0.05% Tween-20, and 1% BSA) for 1 h, followed by secondary antibody (horse anti-mouse HRP-conjugated IgG, Cell Signaling Technology), diluted 1:5000 in antibody buffer, for 1.5 h at room temperature. After washing three times with 1× HBS-T and two times with 1× HBS, 200 μL HRP substrate (SigmaFast OPD) was added per well. The plates were incubated at room temperature, and the absorbance was read at 450 nm after 15 min and 30 min using a SpectraMax 340 PC plate reader (Molecular Devices). For dose-response measurements, absorption values were corrected for unspecific binding of Hsp90 to immobilized GST, and the raw data were fit to equation (1), where f(I) is the fraction of complex remaining, [I] is the concentration of the inhibitor, IC_50_ is the half maximal inhibitory concentration against Hsp90-p50^Cdc37^ complex formation, *n* is the Hill slope coefficient, *min* is the signal remaining at high inhibitor concentrations and *max* is the signal of the uninhibited complex. Corrected absorption values (ΔOD) for the uninhibited Hsp90-p50^Cdc37^ complex (unphosphorylated and phosphorylated) were in the range of 0.4–1.0. Data were expressed relative to the respective *max* value of Equation (1).

(1) $$f(I)=\min+\frac{\left(\max-\min\right)}{1+{\left(\frac{\left[I\right]}{IC_{50}}\right)}^{n}}$$

### 3.8. Isothermal Titration Calorimetry (ITC)

Binding of p50^Cdc37^ and small molecules to Hsp90 was analyzed using a MicroCal iTC200 titration calorimeter (GE Healthcare). Compounds or p50^Cdc37^ were titrated into a solution of Hsp90 using the following concentrations: p50^Cdc37^ (200 µM) into Hsp90 (20 µM), nucleotides (600 µM) into Hsp90 (30 µM), inhibitors (300 µM) into Hsp90 (30 µM). Titrations of compounds into p50^Cdc37^ were performed with similar compound/protein ratios. All experiments were conducted in 50 mM HEPES pH 7.5, 10 mM MgCl_2_ at 30 °C. For titration of inhibitors, the buffer contained 5% DMSO. Typically, an initial 0.5 µL injection was followed by 19 injections of 2.2 µL of syringe solution (compound or p50^Cdc37^) into a 200 µL protein solution of Hsp90 constantly stirred at 1000 rpm, and data were recorded for 180 s between injections. Generation of heat due to dilution was determined in separate experiments by diluting protein into buffer and subtracting these as blank values for each injection. Corrected heat values were fitted using a nonlinear least squares curve-fitting algorithm (Microcal Origin 7.0) to obtain the stoichiometry (n), binding constants (*K*_a_, *K*_d_), and change in enthalpy for each enzyme-ligand interaction (ΔH).

## 4. Conclusions

**Figure 7:**
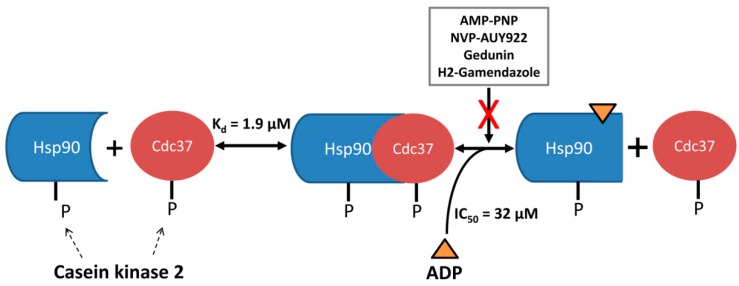
Graphical illustration of the major findings of this work.

Targeted disruption of the Hsp90-p50^Cdc37^ complex by PPI inhibitors has emerged as an alternative strategy to treat diseases characterized by aberrant Hsp90 activity. However, reported PPI inhibitors of Hsp90-p50^Cdc37^ such as gedunin and H2-GMZ have not been validated by direct binding studies or co-crystal structures. In this study, we applied three methods to comprehensively evaluate the effect of selected Hsp90 inhibitors and nucleotides on complex formation, and the influence of phosphorylation by CK2. We found that ADP is an effective inhibitor of the phosphorylated complex with an IC_50_ value of 32 µM, while ATPase and PPI inhibitors of Hsp90 were inactive. Notably, previous studies using yeast Hsp90 showed that the ATPase inhibitor geldanamycin did not influence complex formation with human p50^Cdc37^ or yeast Cdc37 protein [39]. The data suggest that phosphorylation by CK2 primes the complex for dissociation upon interaction with ADP, or upon hydrolysis of bound ATP (Figure 7). Hsp90 harbours multiple phosphorylation sites for CK2 while p50^Cdc37^ is phosphorylated at a single site, but the structural consequences of phosphorylation for the complex are unknown. Current structural information is limited to the complex of the N-terminal domain of yeast Hsp82 with the C-terminal domain of p50^Cdc37^ [30]. Our data indicate that regions beyond the N-terminal domain of Hsp90 participate and influence the complex, possibly the charged linker which harbours two CK2 phosphorylation sites. Hsp90 has been proposed as an ATP sensor, regulating the stability of client proteins in response to the ATP/ADP ratio [40]. The differential activity of ADP over ATP (10-fold difference in IC_50_ values) suggests that a drop in the ATP/ADP ratio causes dissociation of the complex, probably followed by the degradation of client proteins [10].

Our findings conflict previous reports from GST pull-down experiments, in which ADP did not exert inhibitory activity against the Hsp90β-p50^Cdc37^ complex [26]. However, 1:1 stoichiometry of both partner proteins was not achieved in these studies, suggesting that the proteins used or the experimental conditions applied were suboptimal for the complete *in vitro* reconstitution of the complex. Gedunin and H2-GMZ were proposed as inhibitors of the Hsp90-p50^Cdc37^ complex based on co-immunoprecipitation experiments with SkBr3 whole cell lysates and proteolytic fingerprinting using rabbit reticulocyte lysate [29]. It is possible that the effects observed in these studies were caused by Hsp90 partner proteins other than p50^Cdc37^ or by post-translational modifications other than phosphorylation by CK2. The approach applied herein provides robust assays for the biochemical evaluation of potential effectors of the Hsp90-p50^Cdc37^ complex, such as phosphorylation by a kinase or the interaction with small molecules. Therefore, the results from this work may serve drug development campaigns towards the identification and validation of compounds with potential as PPI inhibitors of Hsp90.
